# Case Report: New presentation of CLIFAHDD syndrome with a novel variant in the NALCN gene and a literature review

**DOI:** 10.3389/fped.2024.1370790

**Published:** 2024-05-30

**Authors:** Yi Chen, Xiaotong Xia, Yiwen Zhang, Li Gao, Chenyiyi He, Jianguo Cao

**Affiliations:** Department of Rehabilitation Medicine, Shenzhen Children’s Hospital, Shenzhen, China

**Keywords:** CLIFAHDD syndrome, NALCN gene, missense variant, hypotonia, developmental delay, genetic testing

## Abstract

**Background:**

Congenital contractures of the limbs and face, hypotonia, and developmental delay (CLIFAHDD) syndrome (OMIM #616266) is an autosomal dominant hereditary disease that can lead to the congenital contracture of the limbs and face, hypotonia, and developmental delay. In addition, it may result in growth retardation and present various clinical symptoms, such as brain atrophy, a small pituitary gland, musculoskeletal abnormalities, abnormal breathing, abdominal hernia, and abnormal facial features. Herein, we describe a novel *de novo* missense genetic variant in the sodium leak channel, non-selective (NALCN) gene that is associated with CLIFAHDD syndrome.

**Case description:**

This study describes a patient with varus deformities in both feet, deviation of the ulnar side of the fingers, and severe hypotonia. This patient was subsequently confirmed to have CLIFAHDD syndrome through genetic testing, which also revealed a novel missense *de novo* genetic variant in the NALCN gene (c.3553G > A, p.Ala1185Thr).

**Conclusions:**

Our findings further enrich the known variant spectrum of the NALCN gene and may expand the range of clinical options for treating NALCN-related disorders.

## Introduction

Congenital contractures of the limbs and face, hypotonia, and developmental delay (CLIFAHDD) syndrome is an autosomal dominant genetic disease characterized by congenital limb and facial contractures, hypotonia, and developmental delay (1). The sodium leak channel, non-selective (NALCN) gene is located at 13q32.3–q33.1 and consists of 45 exons, 43 of which are protein-coding and 2 of which are non-coding. More than 40 *de novo* variants have been described to date (2). NALCN encodes for a sodium leak channel that is widely expressed in the membrane of nerve cells, where it regulates the resting membrane potential and subsequently neuron excitability.

The NALCN channel comprises four homologous pore-forming domains (I–IV), each of which comprises six transmembrane fragments (S1–S6). The pore-forming regions S5-P loop-S6 contains the Na+ selective filter EEKA (2). NALCN variants are associated with two neurodevelopmental disorders: CLIFAHDD syndrome (OMIM #616266) and infantile hypotonia with psychomotor retardation and characteristic facies (IHPRF; OMIM #615419). To the best of our knowledge, up to 40 patients with CLIFAHDD syndrome have been reported, and all had *de novo* pathogenic variants in the NALCN gene (2). *De novo* missense pathogenic variants in or near the S5 and S6 pore-forming segments of NALCN result in gain-of-function, and they have been previously implicated as the genetic cause of CLIFAHDD (3, 4). The phenotypic spectrum described by the literature suggests that there is wide variability in clinical phenotype.

A new NALCN variant, c.3553G > A (p. Ala1185Thr), is reported in this study. Our findings further enrich the known variation spectrum of the NALCN gene and may expand the clinical scope of NALCN-related diseases and their treatment.

## Clinical case presentation

A 9-month-old boy was brought to the Department of Rehabilitation Medicine of Shenzhen Children's Hospital in August 2023 due to “low muscle tension in the limbs and neck, and the inability to turn over and sit up on his own.” He was the mother’s only child, delivered naturally at full-term, and had no history of neonatal asphyxia. His birth weight was 2,800 g, and he had no history of convulsions, hepatitis, tuberculosis, trauma, or other infectious diseases. He had no history of food or drug allergies. His parents were young and healthy and had no family history of genetic metabolic diseases.

**Figure 1:**
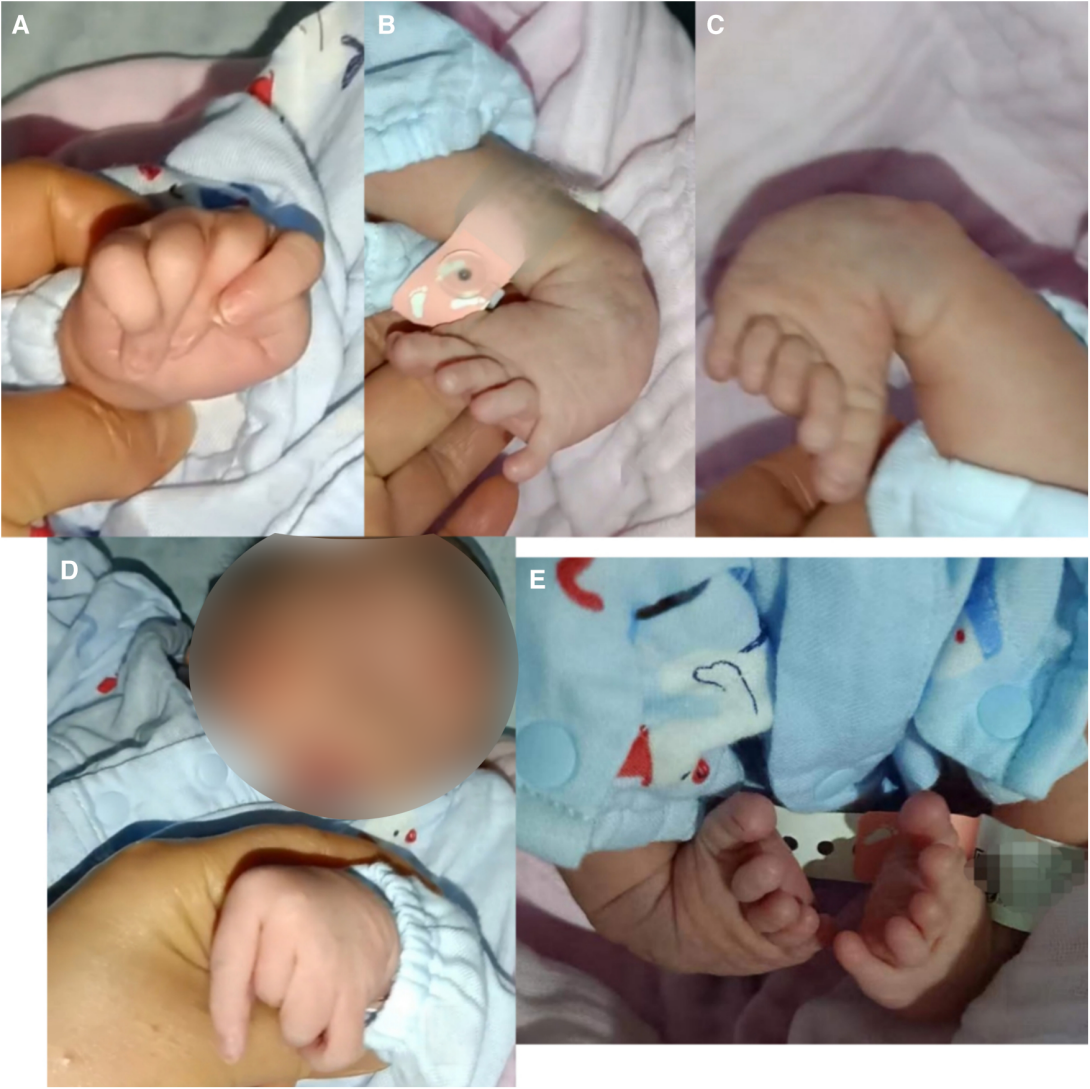
Appearance of the proband. (**A,D**) Congenital contractures of the face and limbs (broad nasal bridge and ulnar deviation). (**B,C,E**) Deformity of the foot (varus deformity).

The boy was born with varus deformity and ulnar deviation of the fingers (see Figure 1). Since his 26th day of life, he has undergone four intermittent plaster corrective operations at a hospital in Guangdong. At 70 days of age, he was treated with percutaneous loosening and Ponseti plaster fixation of the bilateral Achilles tendons for his congenital equine varus foot; since then, he has worn orthopedic braces for at least 3 h every day. At the age of 5 months, he was given an orthopedic brace at a hospital in Beijing due to his finger contracture deformity.

A physical examination at 9 months showed that the patient's head circumference was 44 cm and that his anterior fontanelle was unenclosed at 1.0 cm × 1.0 cm. His facial features included a broad nasal bridge, large nares, a long philtrum, and micrognathia (see Figure 1). There were no abnormalities in eye distance or the palatal arch, and the palmprints of his hands were normal. No obvious abnormalities were found in the lungs, chest, anus, or external genitalia. However, he had a right inguinal hernia that developed when crying and spontaneously recovered. The muscles of the patient's limbs and torso were flaccid, and he had developed hypotonia. Neither the knee reflex nor the Achilles tendon reflex was elicited. He had no sleep problems, breathing problems, or problems when exercising.

Thyroid function and myocardial enzymes showed no abnormalities. The results of a blood test performed on the patient were as follows: myoglobin, 37 ng/ml; creatine kinase, 171 IU/L; lactate dehydrogenase, 280 IU/L; aspartate aminotransferase, 31 IU/L; and creatine kinase–MB, 7.42 ng/ml. The patient's brain magnetic resonance imaging (MRI) and electroencephalogram data were normal, and no characteristic lesions were found via electromyography. A color Doppler ultrasound revealed a patent foramen ovale in his heart (1.4 mm). Due to his low muscle tone, other hospitals suspected that the patient had spinal muscular atrophy (SMA); as a result, he underwent an SMA genetic test via the multiplex ligation-dependent probe amplification (MLPA) method. The results showed rsa 5q13.2 (SMN1 exon 7) × 4 and (SMN1 exon 8) × 4 (these are known genetic variants in the SMN1 gene located on chromosome 5q13.2). The variant involved a duplication of exons 7 and 8 of the SMN1 gene; “×4” indicates that each of these duplications was present in four copies. At the age of 9 months, the child had low muscle tension in the limbs and neck, an unstable vertical head position, and could not turn over or sit up on his own. The Bailey Infant Development Scale Assessment: Intelligence revealed the patient's intelligence to be consistent with that expected at 6 months of age, and his developmental index was less than 50, indicating developmental delay. His Peabody Motor Developmental Scale (PMDS) score was low, with a gross motor development score of 62. His fine motor development was at a medium to low level.

We conducted genetic testing to determine the cause of the patient's overall developmental delay and hypotonia. After obtaining approval from the Medical Ethics Committee of the hospital and informed consent from the patient's parents, peripheral blood samples were collected from the child and his parents and subsequently sent to Suzhou Sai fu Medical Laboratory for testing.

## Whole-exome sequencing

Blood samples were collected from the patient and his parents, and genomic DNA was extracted. The genomic DNA was first sheared into fragments and purified, then subsequently captured using the XGen Exome Research Panel (IDT, USA). Finally, the libraries were sequenced on a NovaSeq 6000 Sequencing platform. After removing low-quality reads and adaptors, the paired-end clean reads were mapped to the human reference genome (GRCh38/hg38) using the Burrows–Wheeler Aligner (BWA). Variant calling was performed via the Genome Analysis Toolkit (GATK). Variants located in exons or classical alternative splicing regions with a low frequency (<0.01) in public databases were obtained for further annotation with ANNOVAR. The pathogenicity of the variants was classified according to the American Society of Medical Genetics and Genomics (ACMG) guidelines.

**Figure 2:**
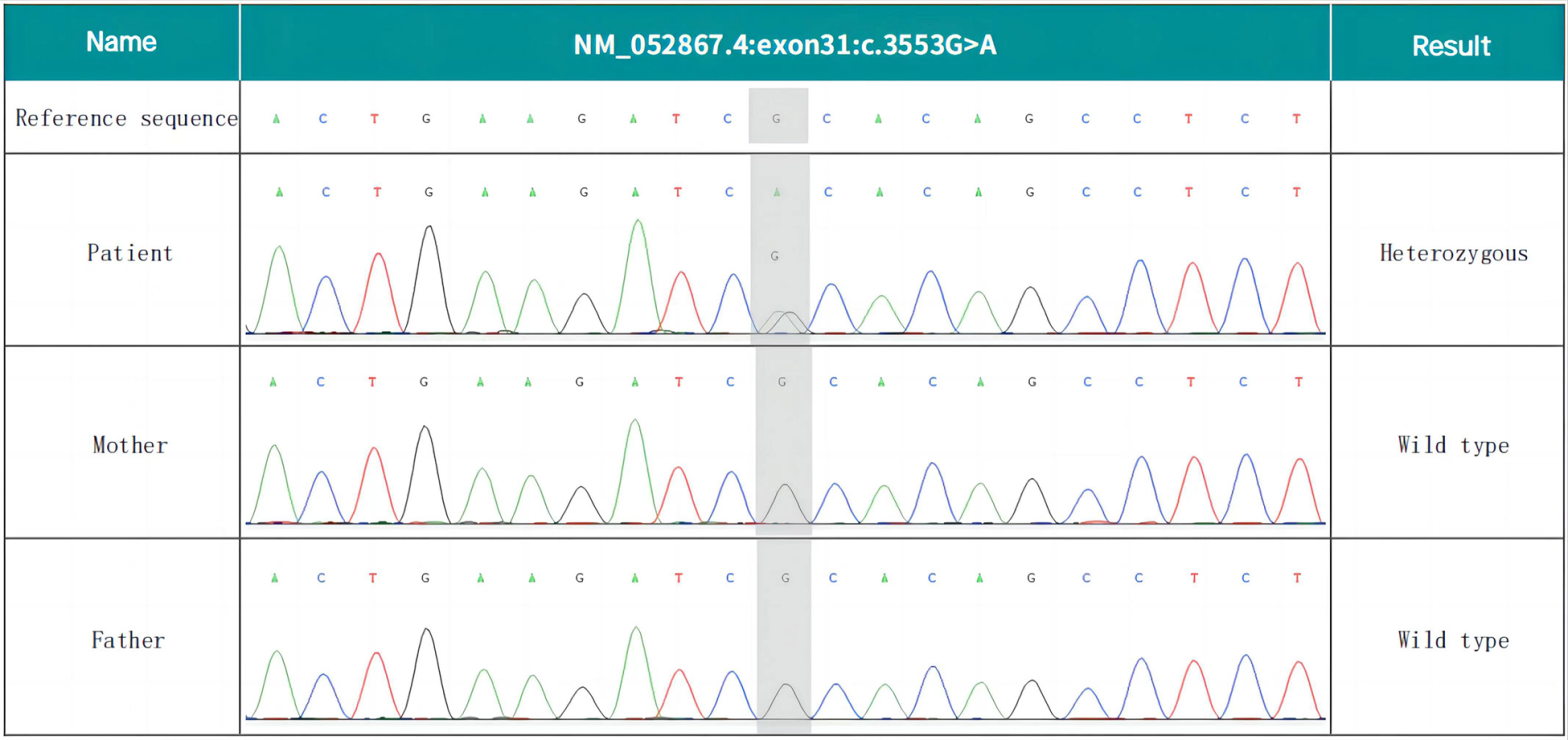
*De novo* NALCN variant of the proband. Whole-exome sequencing revealed the c.3553G > A (p. Ala1185Thr) missense variant.

Whole-exome sequencing revealed that the patient's NALCN gene had a c.3553G > A (p. Ala1185Thr) heterozygosity variation, which was determined to be a new variation through a comparison with the results of parental DNA Sanger sequencing (see Figure 2). The pathogenicity of this variant was analyzed according to the “Interpretation Criteria and Guidelines of Gene Sequence Variation” formulated by the ACMG and the American Society of Molecular Pathology (AMP) (5).

The following evidence about the variant was determined through this analysis: (1) the variant was verified as novel by familial analysis, with the supporting evidence rated as PS2_Moderate; (2) the variant was not recorded in the 1,000-person genome (1000G), human exon database (ExAC), or population genome variant frequency database (gnomAD), and the evidence was rated as PM2_Supporting; (3) the Z-score of this missense variant in gnomAD was 4.96 (ref: 3.09), indicating that the gene had a high intolerance for missense mutations and that it complied with the standards of PP2, as outlined in the ACMG guidelines; (4) the variant was predicted to be harmful using SIFT (a prediction software), Polyphen, Mutation Taster, and other software, and the evidence was rated as PP3; and (5) the patient's clinical symptoms were found to be highly consistent with the disease associated with this gene, and the evidence was rated as PP4.

In summary, according to the above evidence, the missense variant of the NALCN gene, c.3553G > A (p. Ala1185Thr), was assessed as a suspected pathogenic variant (PS2_modem + PM2_supporting + PP2 + PP3 + PP4).

## Discussion

CLIFAHDD syndrome was first detected and defined by Chong et al. in 2015 (1). The NALCN gene (Locus MIM: 611549) is the only pathogenic gene associated with CLIFAHDD syndrome. Up to 40 cases of this disease have been reported worldwide, all with missense variants (2). The clinical spectrum of these cases is reviewed here. Previous reports (6) have indicated that facial contractures, arthrogryposis, joint contractures, hypotonia, and developmental delay (including cognitive delay, motor delay, and speech delay) are among the most prevalent manifestations. We reviewed the literature and found that 70%–80% of the patients had typical facial features (i.e., a broad nasal bridge, large nares, pursed lips, and deep nasolabial folds), 77% exhibited ulnar deviation of the fingers, and 54% had clubfoot. In addition, 60%–80% of patients had developmental delays, and 69% had different degrees of brain atrophy. The primary manifestation in our patient was abnormal limbs (ulnar deviation of both hands, adduction of the thumb, talipes equinovarus, inguinal hernia, hypotonia, and motor retardation), which is consistent with the clinical features previously reported in the literature.

Notably, before our patient was admitted to the hospital, his hand and foot deformities had been corrected; the main problems he still exhibited were severe hypotonia and developmental delays, which differed from the classic CLIFAHDD phenotype. His brain MRI and electroencephalogram results showed no abnormalities; therefore, there was a high likelihood of misdiagnosis. Thus, clinicians should pay more attention to the differential diagnosis of diseases with similar symptoms.

There are various causes of congenital contractures of the limbs and face in children. This case is induced by a new NALCN variant. In cases of congenital limb and face contracture, attention should be paid to the identification of distal arthrogryposis (DA). DA is an autosomal recessive genetic disease characterized by the congenital contracture of the limbs and face alongside normal cognitive development (7). DA is divided into type 1 (DA1) and type 2 (DA2A and DA2B). Among them, DA2A (Freeman–Sheldon) is caused only by the MYH3 gene variant, whereas DA1 and DA2B (Sheldon–Hall syndrome) can be caused by variants in the TPM2, TNNT3, TNN12, and MYH3 genes (8). When a patient has special facial and limb deformities, these genes should be the focus of analysis, and the cognitive development of patients should be evaluated to determine whether there is cognitive impairment. Clinical characteristics are the main basis for distinguishing CLIFAHDD syndrome from DA disease, and a differential diagnosis should be combined with clinical symptoms.

It should also be confirmed that the patient does not have SMA due to muscle relaxation and severe hypotonia. SMA is an autosomal recessive disorder of the nervous system in which SMN1 gene variants lead to progressive atrophy of skeletal muscle and weakness of the limbs (9). SMA manifests as severe hypotonia in infancy. In this case, a SMA genetic diagnosis was performed, and SMA was excluded. Notably, the copy number of exons 7 and 8 of the SMN1 gene in this patient was 4, and copy number duplication is very rare in clinical practice. Some scholars believe that SMN1 duplication may be related to susceptibility to amyotrophic lateral sclerosis (ALS). The literature indicates that abnormal repeats of the SMN1 gene can increase the risk of sporadic ALS by 1.76–2.03 times; however, the specific mechanism involved is not yet clear (10). Further research is needed to determine whether the abnormal repeats of exons 7 and 8 of the SMN1 gene in this patient increased his risk of developing motor system diseases.

Furthermore, the patient had symptoms of hypotonia, growth delay, and abnormal NALCN channels, all of which should be distinguished from IHPRF syndrome. The NALCN channel is an ion channel complex composed of many proteins, including UNC80, UNC79, G protein-coupled receptor (GPCR), and NALCN; in this complex, NALCN forms the channel pore, and UNC80 and UNC79 contribute to the stability of the channel body and neuronal localization (11). GPCRs modulate NALCN activity (11). IHPRF can be divided into two types: IHPRF1 (OMIM 615,419) is an autosomal recessive genetic disease caused by a biallelic variant of NALCN; and IHPRF2 (OMIM 616,801) is a disease caused by a biallelic variant of UNC80 (12, 13). The case presented here was differentiated from IHPRF1. IHPRF1 and CLIFAHDD are both characterized by growth retardation and dystonia; however, IHPRF1 syndrome (14) is rare in distal joint contracture and is different from CLIFAHDD in heredity. Given the combined clinical manifestations and genetic data of this patient, IHPRF1 syndrome was not considered.

According to other reports (3, 8), patients with IHPRF and patients with CLIFAHDD exhibit several common clinical symptoms, such as developmental delay, hypotonia, respiratory defects, and constipation. Furthermore, NALCN function is differentially altered between these conditions. NALCN channel function is lost in patients with IHPRF variants while CLIFAHDD variants are gain-of-function ones. Both are expected to impact on cell excitability. The CLIFAHDD variant may decrease cell excitability (3). Thus, the symptoms are similar to those observed in patients with the IHPRF loss-of-function phenotype. Therefore, some experts have speculated that inhibiting the activity of some NALCN channels may be beneficial for treating CLIFAHDD. However, further studies are needed to explore this phenomenon and the detailed mechanisms involved.

## Conclusion

In conclusion, we described the CLIFAHDD phenotypes associated with a novel variant in the NALCN gene, c.3553G > A (p. Ala1185Thr), in a Chinese infant. Our findings further expand the known variant spectrum of the NALCN gene and may expand the clinical range of treatment of NALCN-related disorders, providing new data for research on CLIFAHDD syndrome. We listed the diseases with symptoms similar to those of CLIFAHDD and conducted a differential analysis to provide clinicians with broader insight into diagnosis and treatment, which may reduce the incidence of misdiagnosis.

## Data Availability

The original contributions presented in the study are included in the article/Supplementary Material, further inquiries can be directed to the corresponding author.

## References

[1] Chong JX McMillin MJ Shively KM Beck AE Marvin CT Armenteros JR De novo mutations in NALCN cause a syndrome characterized by congenital contractures of the limbs and face, hypotonia, and developmental delay. Am J Hum Genet. (2015) 96(3):462–73. 10.1016/j.ajhg.2015.01.003 25683120 PMC4375444

[2] Monteil A Guérineau NC Gil-Nagel A Parra-Diaz P Lory P Senatore A. New insights into the physiology and pathophysiology of the atypical sodium leak channel NALCN. Physiol Rev. (2024) 104(1):399–472. 10.1152/physrev.00014.2022 37615954

[3] Bouasse M Impheng H Servant Z Lory P Monteil A. Functional expression of CLIFAHDD and IHPRF pathogenic variants of the NALCN channel in neuronal cells reveals both gain- and loss-of-function properties. Sci Rep. (2019) 9(1):11791. 10.1038/s41598-019-48071-x 31409833 PMC6692409

[4] Kschonsak M Chua HC Noland CL Weidling C Clairfeuille T Bahlke OØ Structure of the human sodium leak channel NALCN. Nature. (2020) 587(7833):313–8. 10.1038/s41586-020-2570-8 32698188

[5] Richards S Aziz N Bale S Bick D Das S Gastier-Foster J Standards and guidelines for the interpretation of sequence variants: a joint consensus recommendation of the American College of Medical Genetics and Genomics and the Association for Molecular Pathology. Genet Med. (2015) 17(5):405–24. 10.1038/gim.2015.30 25741868 PMC4544753

[6] Vivero M Cho MT Begtrup A Wentzensen IM Walsh L Payne K Additional de novo missense genetic variants in NALCN associated with CLIFAHDD syndrome. Clin Genet. (2017) 91(6):929–31. 10.1111/cge.12899 28133733

[7] Al-Sayed MD Al-Zaidan H Albakheet A Hakami H Kenana R Al-Yafee Y Mutations in NALCN cause an autosomal-recessive syndrome with severe hypotonia, speech impairment, and cognitive delay. Am J Hum Genet. (2013) 93(4):721–6. 10.1016/j.ajhg.2013.08.001 24075186 PMC3791267

[8] Hashemi B Huntsman RJ Li H Zhang D Xi Y. New presentation of CLIFAHDD syndrome with a novel variant in NALCN gene: a report of a rare case. Clin Case Rep. (2023) 11(7):e7647. 10.1002/ccr3.7647 37469362 PMC10352546

[9] Ribero VA Marti Y Batson S Mitchell S Gorni K Gusset N Systematic literature review of the natural history of spinal muscular atrophy: motor function, scoliosis, and contractures. Neurology. (2023) 101(21):e2103–13. 10.1212/WNL.0000000000207878 37813581 PMC10663020

[10] Blauw HM Barnes CP van Vught PW van Rheenen W Verheul M Cuppen E SMN1 gene duplications are associated with sporadic ALS. Neurology. (2012) 78(11):776–80. 10.1212/WNL.0b013e318249f697 22323753 PMC3304946

[11] Aoyagi K Rossignol E Hamdan FF Mulcahy B Xie L Nagamatsu S A gain-of-function mutation in NALCN in a child with intellectual disability, ataxia, and arthrogryposis. Hum Mutat. (2015) 36(8):753–7. 10.1002/humu.22797 25864427

[12] Liao Z Liu Y Wang Y Lu Q Peng Y Liu Q. Case report: a de novo variant in NALCN associated with CLIFAHDD syndrome in a Chinese infant. Front Pediatr. (2022) 10:927392. 10.3389/fped.2022.927392 35911839 PMC9326163

[13] Bramswig NC Bertoli-Avella AM Albrecht B Al Aqeel AI Alhashem A Al-Sannaa N Genetic variants in components of the NALCN-UNC80-UNC79 ion channel complex cause a broad clinical phenotype (NALCN channelopathies). Hum Genet. (2018) 137(9):753–68. 10.1007/s00439-018-1929-5 30167850 PMC6671679

[14] Angius A Cossu S Uva P Oppo M Onano S Persico I Novel NALCN biallelic truncating mutations in siblings with IHPRF1 syndrome. Clin Genet. (2018) 93(6):1245–7. 10.1111/cge.13162 29399786

